# Research on Speech Synthesis Based on Mixture Alignment Mechanism

**DOI:** 10.3390/s23167283

**Published:** 2023-08-20

**Authors:** Yan Deng, Ning Wu, Chengjun Qiu, Yan Chen, Xueshan Gao

**Affiliations:** 1 School of Computer, Electronics and Information, Guangxi University, Nanning 530004, China; 2113391006@st.gxu.edu.cn; 2 Key Laboratory of Beibu Gulf Offshore Engineering Equipment and Technology, Beibu Gulf University, Qinzhou 535011, China; 3 College of Mechanical Naval Architecture and Ocean Engineering, Beibu Gulf University, Qinzhou 535011, China; 4 Guangxi Key Laboratory of Ocean Engineering Equipment and Technology, Qinzhou 535011, China

**Keywords:** speech synthesis, acoustic signal processing, mixture attention mechanism, deep learning

## Abstract

In recent years, deep learning-based speech synthesis has attracted a lot of attention from the machine learning and speech communities. In this paper, we propose Mixture-TTS, a non-autoregressive speech synthesis model based on mixture alignment mechanism. Mixture-TTS aims to optimize the alignment information between text sequences and mel-spectrogram. Mixture-TTS uses a linguistic encoder based on soft phoneme-level alignment and hard word-level alignment approaches, which explicitly extract word-level semantic information, and introduce pitch and energy predictors to optimally predict the rhythmic information of the audio. Specifically, Mixture-TTS introduces a post-net based on a five-layer 1D convolution network to optimize the reconfiguration capability of the mel-spectrogram. We connect the output of the decoder to the post-net through the residual network. The mel-spectrogram is converted into the final audio by the HiFi-GAN vocoder. We evaluate the performance of the Mixture-TTS on the AISHELL3 and LJSpeech datasets. Experimental results show that Mixture-TTS is somewhat better in alignment information between the text sequences and mel-spectrogram, and is able to achieve high-quality audio. The ablation studies demonstrate that the structure of Mixture-TTS is effective.

## 1. Introduction

Speech synthesis has gained great progress with the introduction of deep learning, and many advanced acoustic models and vocoders have emerged, which synthesize audio with far better quality than the previous traditional speech synthesis models [1,2,3,4]. Speech synthesis is a one-to-many mapping generation task that processes the input text to synthesize high-quality audio samples. The deep learning-based speech synthesis model simplifies the complex text processing such as text normalization and phonemization and then simplifies the TTS training scheme into two stages. The acoustic model, as the first stage, converts the input phoneme sequences into acoustic features (e.g., mel-spectrogram). The vocoder, as the second stage, takes the generated acoustic features as input conditions and synthesizes the speech waveform [5,6,7]. The acoustic model and vocoder are trained separately as two independent tasks, which will result in a complex TTS model-training channel. Several studies have focused on end-to-end speech synthesis models, where speech waveforms can be obtained by inputting text sequences only, simplifying the training scheme and optimizing the acoustic feature mismatch problem [8,9,10]. In this paper, we focus on a speech synthesis model with a two-stage training scheme.

Autoregressive models based on neural networks have shown the ability to generate high-quality audio [11,12,13]. However, since autoregressive models use frame-by-frame inference, they usually face the problem of slow training and inference. To improve the speed of a model-synthesized audio, a large amount of research has been focused on non-autoregressive models. Several studies have already focused on obtaining alignment information between the text and mel-spectrogram. Some non-autoregressive models use a teacher model-based knowledge distillation approach [14], while others focus on an external alignment tool (Montreal forced alignment, MFA) [8] or use a normalized flow and dynamic programming method to directly search for the most likely monotonic alignment information between the text and mel-spectrogram [9,15]. In addition, speech synthesis has introduced some other generative models that have also achieved very good performance. Flow-based models are found in [15,16,17], variational autoencoder (VAE)-based models are listed in [17,18], generative adversarial network (GAN)-based models are presented in [19], and diffusion process-based models are described in [20,21,22,23].

To further improve the quality of synthesized audio, this paper proposes Mixture-TTS, a non-autoregressive speech synthesis model. Mixture-TTS introduces the linguistic encoder based on the mixture alignment mechanism in PortaSpeech [17]. On top of this, additional pitch and energy predictors are added to optimize the rhythmic information of the synthesized audio. To optimize the reconstructability of the mel-spectrogram, Mixture-TTS introduces a post-net structure based on a five-layer 1D convolution network [12]. Mixture is connected to the output of the post-network through the residual network after the decoder and passed into the HiFi-GAN vocoder [6]. We evaluate the Mixture-TTS on the AISHELL3 [24] and LJSpeech [25] datasets. Both datasets are open-source datasets that have been professionally recorded and processed to ensure that the model reproduces realistic speech information. Experimental results show that Mixture-TTS achieves good results in terms of synthesizing audio quality, predicted mel-spectrogram, and attention alignment.

## 2. Background

In this section, we will introduce the FastSpeech2, and then introduce the HiFi-GAN vocoder used.

### 2.1. FastSpeech2

**Figure 1:**
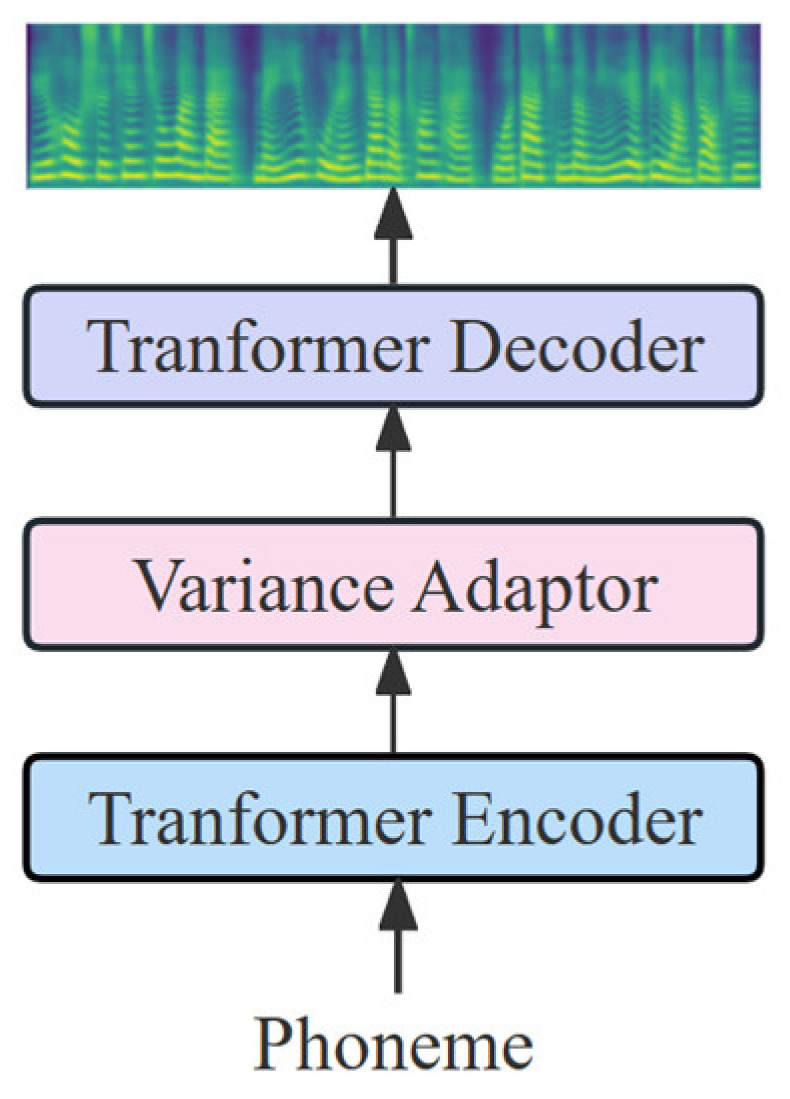
The overall architecture for FastSpeech2.

FastSpeech2 is a non-autoregressive TTS model based on neural networks, and with a structure as that shown in Figure 1. FastSpeech2 consists of an encoder, a variance adaptor, and a decoder. Both the encoder and decoder use the feed-forward transformer (FFT) [26] consisting of a series of self-attention layers and a 1D convolution network as the basic structure. To add sufficient variance information in training to improve the expressiveness of the synthesized audio, FastSpeech2 introduces the variance adaptor, which includes duration, pitch, and energy predictor. In addition, FastSpeech2 uses the MFA to obtain the duration of the phoneme.

FastSpeech2 receives the phoneme sequences, the encoder encodes the phoneme sequences into phoneme hidden states, and the variance adaptor provides phoneme duration, pitch, and energy variance information to the phoneme hidden states. The decoder receives the phoneme hidden states with additional variance information and decodes them into output sequences to obtain the mel-spectrogram.

### 2.2. HiFi-GAN

The HiFi-GAN vocoder consists of one generator and two discriminators. The backbone of the generator is a convolution neural network (CNN) that takes the mel-spectrogram as an input condition. The generator continuously up-samples the mel-spectrogram until the length of the output sequences is the same as the time domain resolution of the original waveform. HiFi-GAN introduces multi-receptive field (MRF) fusion module in the generator to fuse features of different scales together, thus improving the perceptual capability and stability. Specifically, the MRF module uses multiple convolution kernels to extract features at different scales, fuse the features at different scales, and generate the final output. The MRF module can better handle the periodic information in the speech signal and improve the naturalness and fluency of the synthesized speech. The discriminators are multi-period discriminator (MPD) and multi-scale discriminator (MSD), respectively.

MPD uses multiple sub-discriminators to process the periodic and non-periodic components of the speech signal separately, modeling the periodic patterns in speech to improve the sample quality. This design approach can optimize the periodic information in the speech signal and improve the naturalness and fluency of the synthesized speech. HiFi-GAN introduces the MSD module, which is able to continuously evaluate the speech sequences predicted by the generator for real/fake classification.

## 3. Mixture-TTS

In this section, we introduce the design motivation of Mixture-TTS, and then we introduce the Mixture-TTS architecture and the loss function composition.

### 3.1. Motivation

**Figure 2:**
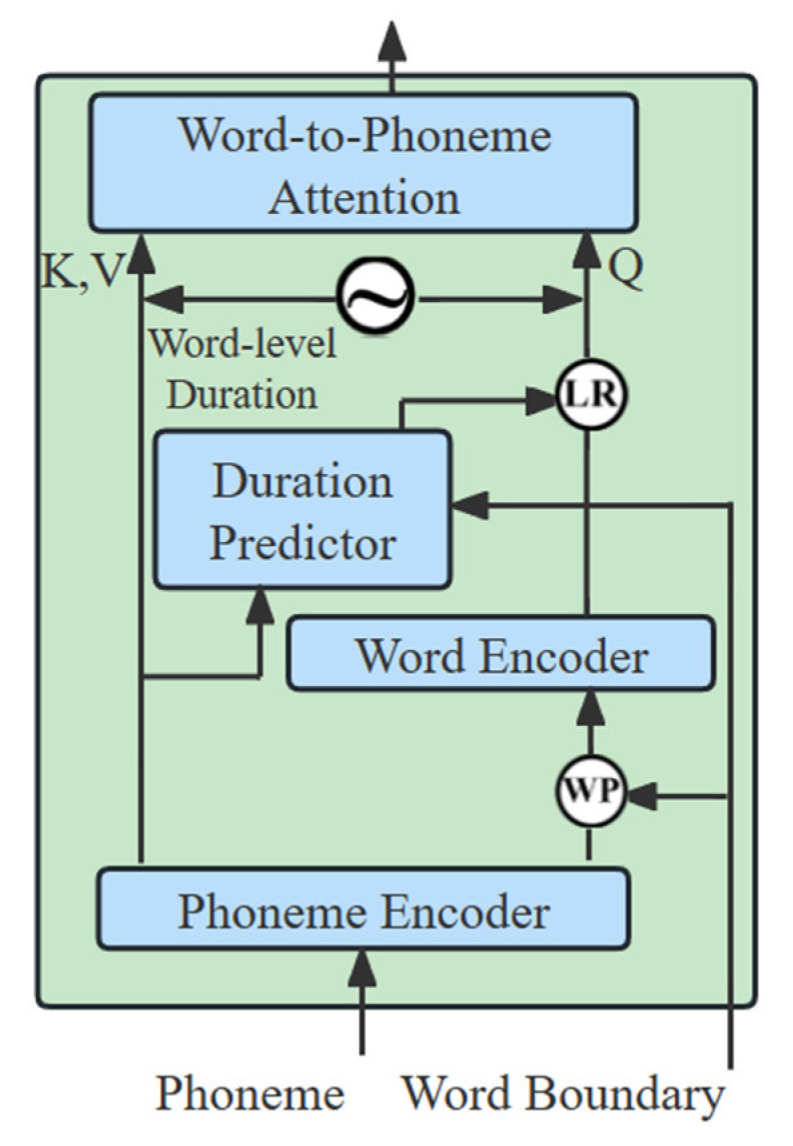
Linguistic encoder architecture for PortaSpeech.

FastSpeech2 uses the MFA to obtain the duration of phonemes, which causes phoneme blurring problems during phoneme alignment. The main reason is that there is no clear boundary between different phonemes in mel-spectrogram. Based on this, PortaSpeech designs a linguistic encoder based on a mixture alignment mechanism. Linguistic encoder uses soft alignment for phonemes and hard alignment for word-level, which solves the problems caused by the hard alignment of phonemes. Mixture-TTS introduces the same linguistic encoder as in the PortaSpeech, as shown in Figure 2. Based on this, the Mixture-TTS incorporates pitch and energy predictors to optimize the rhythmic information of the synthesized audio.

Tacotron2 is a convolutional neural network-based TTS model that achieved impressive results in real time. Tacotron2 introduces a post-net network based on a five-layer 1D convolution network after the decoder, which is experimentally shown to optimize the reconfiguration capability of the mel-spectrogram. The mel-spectrogram is a very important intermediate feature of the speech synthesis task that directly affects the quality of the final synthesized audio. Therefore, we also optimize the prediction details of the mel-spectrogram. Specifically, we introduce the same post-net network and confirm the validity of the introduction of the post-net network in the Mixture-TTS in the ablation experiment section.

### 3.2. Basic Model Architecture

**Figure 3:**
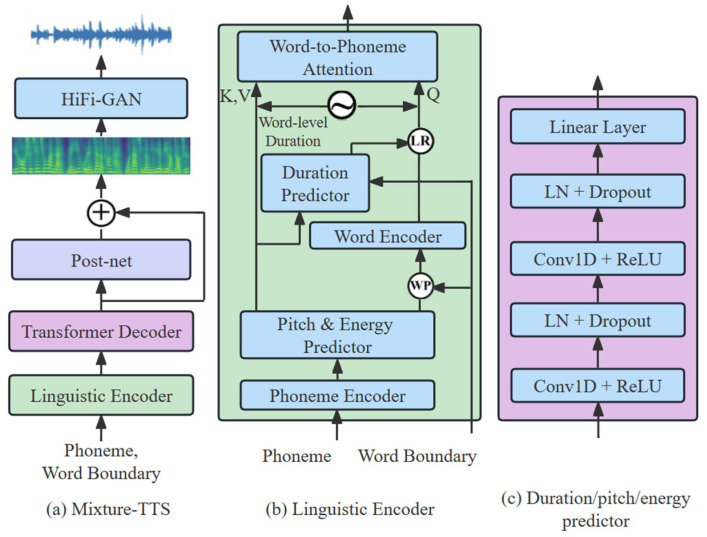
The overall architecture for Mixture-TTS.

As shown in Figure 3a, Mixture-TTS consists of a linguistic encoder, a transformer decoder, and a post-net. In the section, we describe the structure and training loss of the Mixture-TTS in detail.

We add pitch and energy predictors to the original linguistic encoder structure to optimize the rhythmic information of the synthesized audio. Specifically, the pitch and energy information of the real audio is extracted by the pitch and energy predictors in the training stage, which provides enough variance information as an additional input condition in the inference stage. The structure of the linguistic encoder is shown in Figure 3b, where “LR” represents the length regulator, “WP” represents the word-level pooling, and sinusoidal-like symbol represents the relative position encoding [26]. In addition, the pitch and energy predictors and the duration predictor have similar structures as shown in Figure 3c. We pass the phoneme sequences with word boundaries as input conditions to the linguistic encoder. The phoneme encoder converts the input phoneme sequences into phoneme hidden states. Additional variance information is added to the phoneme hidden state via pitch and energy predictors. We apply word-level pooling on the phoneme hidden state to obtain the input conditions for the word encoder, and then average the phoneme hidden states within each word according to the word boundaries. The word encoder receives the phoneme hidden states and then encodes them as the word-level hidden states. In addition, we use a length regulator with word-level duration to extend the word-level hidden states to match the length of the target mel-spectrogram. Finally, the phoneme hidden states and word-level hidden states are encoded by word-level relative position and passed to the word-phoneme attention module.

**Figure 4:**
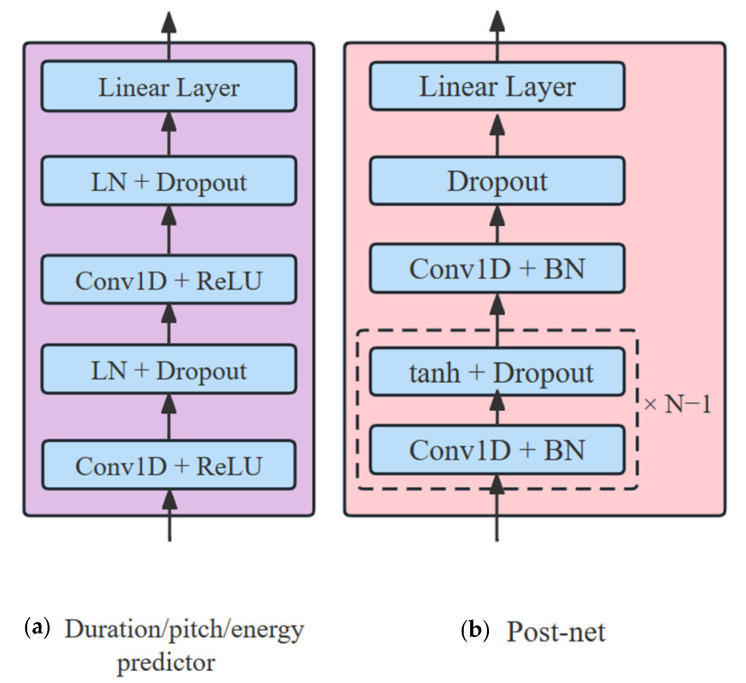
Structure of FFT module and post-net.

The decoder structure is the same as the phoneme encoder and word encoder structures, both of which have the FFT as the basic structure and no longer contain the recurrent neural network structure. We set the number of FFT of phoneme encoder and word encoder to 4, and the number of FFT of decoder to 6. The number of heads in the multi-head attention mechanism are set to 2, the convolution kernel size is set to 9, and the dropout is set to 0.2. The structure of the FFT module is shown in Figure 4a, and the contextual information of the phoneme sequences is obtained using the multi-headed attention mechanism, and then connected to the input phoneme sequences by the residual network [27] after dropout and normalization. Then, the contextual information is fused by a two-layer 1D convolution network and is passed to the next module after dropout and normalization.

Mixture-TTS introduces a post-net to improve the overall mel-spectrogram reconstruction, and connects the decoder output to the post-net output through the residual network. As shown in Figure 4b, the post-net is a five-layer 1D convolution network, each layer consists of 512 number of 5 × 1 convolution kernels, batch normalization (BN) and dropout, accompanied by Tanh activation function except for the last layer. The output of the last layer is linearly mapped to obtain the mel-spectrogram.

### 3.3. Training Loss

To train Mixture-TTS, we use minimizing the overall error $\tau_{total}$ between the ground truth and predicted values:

(1) $$\tau_{total}\;=\;\tau_{mel}\;+\;\tau_{postnet}\;+\;\tau_{duration}\;+\;\tau_{helper}\;+\;\tau_{pitch}\;+\;\tau_{energy}$$

where $\tau_{mel}$ and $\tau_{postnet}$ use mean absolute error (MAE) loss, while $\tau_{duration}$, $\tau_{pitch}$, and $\tau_{energy}$ use mean squared error (MSE) loss. $\;\tau_{helper}$ uses the guided attention loss [28].

## 4. Experiments and Results

We evaluate the Mixture-TTS on the AISHELL3 and LJSpeech datasets and select the FastSpeech2, PortaSpeech, DiffGAN-TTS (*T* = 4), and DiffGAN-TTS (two-stage) as the comparison experiments of the models. The DiffGAN-TTS introduces a GAN-based diffusion model based on FastSpeech2, which reduces the number of denoising steps to 4. Based on this, the DiffGAN-TTS model proposes an active shallow diffusion mechanism, which further reduces the number of denoising steps to 1. DiffGAN-TTS (*T* = 4) denotes the DiffGAN-TTS with 4 denoising steps, and DiffGAN-TTS (two-stage) denotes the DiffGAN-TTS with an active shallow diffusion mechanism applied. In this section, we introduce the dataset and model configuration, and then we describe the evaluation methods and experimental results. Finally, we evaluate the structures introduced by the Mixture-TTS to validate the effectiveness of each structure.

### 4.1. Datasets

We conduct experiments on the AISHELL3 and LJSpeech datasets. The AISHELL3 dataset consists of 88,035 Chinese audio clips recorded by 218 Mandarin speakers, with a total duration of approximately 85 h. For the AISHELL3 dataset, we select 87,011 samples for training. The LJSpeech dataset consists of 13,100 English audio clips from a single speaker, with a total length of approximately 24 h. We select 12,076 samples for training. The validation dataset and test dataset for both datasets are 512 samples. For the two test datasets, we randomly select 150 samples for objective evaluation and 20 samples for subjective evaluation. In addition, the Chinese and English text sequences are converted to phoneme sequences by pypinyin and g2p_en libraries, respectively. We convert the original waveform to a mel-spectrogram at a sampling rate of 22,050 Hz, setting the frame length and hop length to 1024 and 256, respectively.

### 4.2. Model Configuration

We train the Mixture-TTS on one NVIDIA 3060 GPU with batch sizes of 8 and 16 on the AISHELL3 and LJSpeech datasets, respectively. We use the Adam optimizer [29] with parameters set to $\beta_{1}$ = 0.9, $\beta_{2}$ = 0.98, ϵ = $10^{-9}$ and which follows the same learning rate scheme as [8,17]. Mixture-TTS reaches convergence after 640k and 400k steps on the AISHELL3 and LJSpeech datasets, respectively. We use the publicly trained HiFi-GAN vocoder to convert the mel-spectrogram into an audio sample. Other comparison models are evaluated using the github open-source version of the code. We keep the text content consistent across models, exclude other confounding factors, and check only the audio quality. The CUDA version for all experiments is 11.6, and the compilation environment is Python 3.8 with PyTorch version 1.8.0+cu111.

### 4.3. Evaluate

We use structural similarity index (SSIM) [30], mel-cepstral distortion (MCD) [31], and F_0_ root-mean-squared error (F_0_ RMSE) metrics for objective evaluation to measure the quality of synthesized audio. We use dynamic time warping (DTW) [32] in the computation of MCD and F_0_ RMSE to align the generated audio with the real reference audio. In addition, We use the logarithmic method in calculating the F_0_ RMSE value. The SSIM is a measure of image similarity. In the TTS experiment, a higher SSIM indicates that the synthesized mel-spectrogram can restore more realistic details. On the contrary, the lower the MCD and F_0_ RMSE, the better the audio quality to a certain extent. The experimental results of the AISHELL3 dataset are shown in Table 1. Mixture-TTS obtains the best performance for SSIM and MCD evaluation, with 0.514 and 17.065, respectively. We observe that Mixture-TTS obtains an F_0_ RMSE score of 0.769 and does not perform the best in predicting the F_0_ fundamental frequency. Param is a measure of the number of model parameters. On the AISHELL3 dataset, the number of parameters of Mixture-TTS is 31.75M. In addition, RTF is a measure of the speed of model inference. We select 10 generated audio samples for RTF testing, which range in duration and number of words from 3 to 6 s and 8 to 20 characters, respectively. RTF test results on the AISHELL3 dataset are shown in Table 1.

The experimental results of the LJSpeech dataset are shown in Table 2. Mixture-TTS still achieves the best performance in the SSIM and MCD evaluations with 0.508 and 6.635, respectively. In terms of F_0_ RMSE, Mixture-TTS is slightly weaker than the other models compared. The number of parameters of Mixture-TTS on the LJSpeech dataset is 31.75M. We select 10 generated audio samples for RTF testing, which range in duration and number of words from 4 to 6 s and 10 to 20 characters, respectively. RTF test results on the LJSpeech dataset are shown in Table 2. Experimental results on the AISHELL3 and LJSpeech datasets show that Mixture-TTS is somewhat better in alignment information between the text sequences and mel-spectrogram, and is able to achieve high-quality audio. We use one NVIDIA 3060 GPU for training and inference of all models.

We use mean opinion score (MOS) [33] and relative evaluation opinion score (CMOS) [34] metrics for subjective evaluation to measure the quality of synthesized audio. Each audio sample is evaluated by ten native testers, both the AISHELL3 and LJSpeech datasets. We conduct the test in a quiet classroom with all testers wearing a headphone. We break up all samples participating in the test, which means that no labels are given for which the model of the sample is generated from. Testers are asked to carefully score each voice on a scale from 1 to 5, in 0.5 increments, on speech naturalness and accent performance. In the AISHELL3 dataset, experiment results show that Mixture-TTS obtains the notable MOS score of 3.92, which is comparable to the performance of PortaSpeech and better than the other models. The experiment results on the LJSpeech dataset also show that the Mixture-TTS can better solve the one-to-many mapping problem in TTS, and the synthesized audio quality is better. In addition, we use the CMOS to further measure the quality of the synthesized audio. We use FastSpeech2 as the baseline model, and the testers test the audio samples synthesized by other models. As can be seen from Table 1 and Table 2, Mixture-TTS performs comparably better with the PortaSpeech, outperforming the other models, which indicates that the audio quality of Mixture-TTS is better.

### 4.4. Feature Prediction

**Figure 5:**
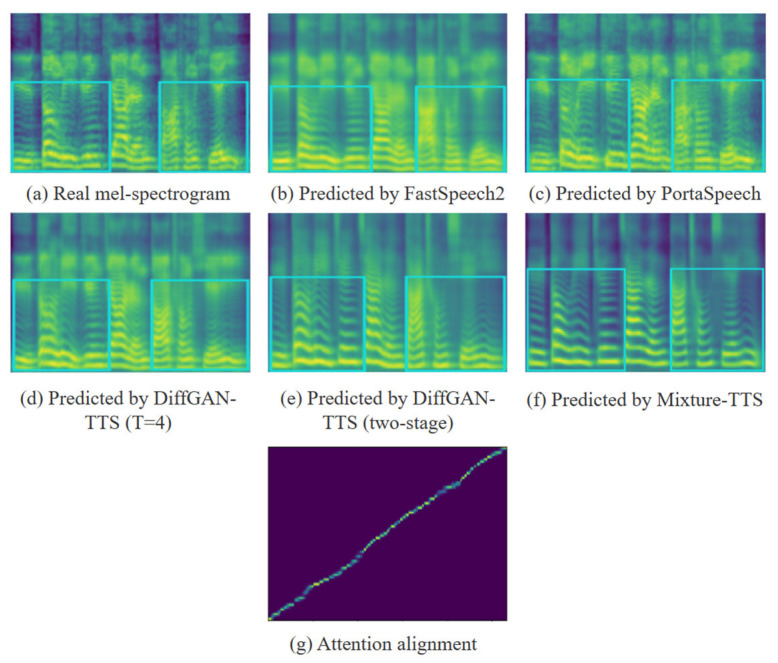
Comparison of feature predictions from different TTS models.

We investigate the mel-spectrogram and attention alignment information on the AISHELL3 dataset. The more details the mel-spectrogram shows, the better the quality of the synthesized audio to a certain extent. The mel-spectrogram synthesized by each model is shown in Figure 5. We can observe the advantage of the Mixture-TTS in predicting the mel-spectrogram details in the band between two adjacent harmonics, unvoiced frames and the low frequency part, which is also corroborated by the experimental results in SSIM and MOS.

For the analysis of the attention alignment graph, the points in the alignment graph are the attention weight values, and the larger the attention weight value is, the brighter the points are. The higher the attention weight, the brighter the dots. The alignment graph can reflect the stability of the speech synthesis model. The clearer the diagonal line formed by the dots, the more accurate the model is in establishing the mapping relationship between phoneme sequences and spectral feature sequences. The brighter the alignment diagram is, the clearer the lines formed, the higher the accuracy of speech synthesis, and the more stable the model is. As shown in Figure 5g, Mixture-TTS exhibits a clear and smooth attentional alignment convergence map, which shows that the Mixture-TTS has a notable ability to align the phoneme sequences with the mel-spectrogram map.

### 4.5. Ablation Studies

We conduct an ablation study on the AISHELL3 dataset of the pitch, energy, post-net, and the residual network by the Mixture-TTS model to demonstrate the effectiveness of each structure. For the ablation study of pitch and energy variance information, we perform a CMOS evaluation. As shown in Table 3, it can be observed that the Mixture-TTS with the removal of pitch leads to a performance degradation in terms of speech quality with a CMOS of −0.168. Removing energy leads to a degraded performance in terms of voice quality of −0.135 for CMOS. Removing pitch and energy will significantly reduce the quality of the generated speech, which is −0.374 for CMOS. The experimental results show that Mixture-TTS introduces pitch and energy variance information in the speech encoder can optimize the rhythmic information of the synthesized audio, thus improving the quality of speech. Mixture-TTS can learn the pitch and energy difference information of real audio in the training stage, thus providing the model with rich difference information in the inference stage.

For the ablation study of the post-net and residual network, we use the SSIM, MCD, F_0_ RMSE, and CMOS to evaluate. We randomly select 150 samples from the AISHELL3 test dataset for an objective evaluation test, and the results are shown in Table 4. Mixture-TTS achieves the best SSIM and MCD values, with a value of 0.769 for the F_0_ RMSE. It can be observed from the CMOS experimental results that the Mixture-TTS outperforms the Mixture-TTS with the removal of the post-net and residual network, which indicates that the Mixture-TTS with the post-net and residual network added has a more competitive mel-spectrogram reconstruction capability and better speech quality.

**Figure 6:**
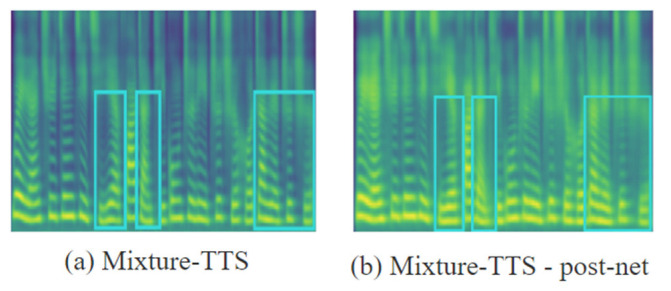
The mel-spectrogram comparison for ablation studies of post-net.

We investigate the mel-spectrogram generated by Mixture-TTS with the post-net structure removed. The predicted mel-spectrogram of Mixture-TTS is given in Figure 6a, and the predicted mel-spectrogram with the post-net structure removed is given in Figure 6b. From Figure 6, it can be observed that the Mixture-TTS without the post-net will lose more details of the mel-spectrogram and the speech quality will be reduced.

## 5. Conclusions

In this paper, we propose Mixture-TTS, a speech synthesis model with a mixture alignment mechanism, which is optimized on the basis of FastSpeech2 and PortaSpeech. Mixture-TTS introduces a linguistic encoder based on a mixture alignment mechanism to solve the boundary blurring problem of the hard alignment of phonemes. On top of this, pitch and energy predictors are added to optimize the rhythmic information of the synthesized audio. In addition, we introduce a post-net structure based on a five-layer 1D convolution network to optimize the reconfiguration capability of the mel-spectrogram.

We evaluate Mixture-TTS on the AISHELL3 and LJSpeech datasets. We use the SSIM, MCD and F_0_ RMSE as objective evaluation metrics, and the MOS and CMOS as subjective evaluation metrics. Mixture-TTS obtains the best SSIM and MCD experimental results under the two datasets, but is slightly weaker in terms of F_0_ RMSE. We conduct an ablation study of the structures added to the Mixture-TTS. The experimental results show the validity of each part of Mixture-TTS. In addition, PortaSpeech already has a flow-based post-net structure to optimize the details of the mel-spectrogram, which is different from the post-net in Mixture-TTS. We will use the existing structure of Mixture-TTS as a basis for further research on other generative models, which is currently a hot topic in the TTS task. Some researchers have focused on fully end-to-end TTS models that obtain speech waveforms from input text sequences only, simplifying the training scheme of traditional TTS models. A full end-to-end model will be our future research plan.

## Figures and Tables

**Table 1:** Model experiment evaluation and model efficiency results on the AISHELL3 dataset.

Model	SSIM (↑)	MCD (↓)	F_0_ RMSE (↓)	Params	RTF	MOS	CMOS
Ground Truth						4.15 ± 0.09	
FastSpeech2	0.494	17.295	0.719	30.87M	0.2276	3.75 ± 0.08	0.000
PortaSpeech	0.512	17.071	**0.715**	24.26M	0.2211	**3.94 ± 0.06**	0.157
DiffGAN-TTS (*T* = 4)	0.509	17.187	0.745	29.04M	0.2231	3.87 ± 0.07	0.148
DiffGAN-TTS (two-stage)	0.511	17.143	0.774	40.25M	0.2243	3.83 ± 0.08	0.146
Mixture-TTS	**0.514**	**17.065**	0.769	31.75M	0.2152	3.92 ± 0.09	0.154

**Table 2:** Model experiment evaluation and model efficiency results on the LJSpeech dataset.

Model	SSIM (↑)	MCD (↓)	F_0_ RMSE (↓)	Params	RTF	MOS	CMOS
Ground Truth						4.21 ± 0.07	
FastSpeech2	0.491	7.007	0.307	30.81M	0.1870	3.82 ± 0.08	0.000
PortaSpeech	0.507	6.662	**0.305**	23.97M	0.1723	**3.99 ± 0.07**	0.192
DiffGAN-TTS (*T* = 4)	0.502	6.724	0.311	29.04M	0.1828	3.94 ± 0.07	0.181
DiffGAN-TTS (two-stage)	0.505	6.689	0.313	40.61M	0.1863	3.91 ± 0.08	0.173
Mixture-TTS	**0.508**	**6.635**	0.316	31.24M	0.1761	3.96 ± 0.09	0.189

**Table 3:** CMOS comparison for Mixture-TTS.

Setting	CMOS
Mixture-TTS	0.000
Mixture-TTS-pitch	−0.168
Mixture-TTS-energy	−0.135
Mixture-TTS-pitch-energy	−0.374

**Table 4:** Post-net and residual network comparisons for Mixture-TTS.

Setting	SSIM (↑)	MCD (↓)	F_0_ RMSE (↓)	CMOS
Mixture-TTS	0.514	17.065	0.769	0.000
Mixture-TTS-residual network	0.511	17.169	0.778	−0.127
Mixture-TTS-post-net	0.508	17.455	0.734	−0.291

## Data Availability

We use the espnet toolkit to calculate MCD and F_0_ RMSE. The espnet toolkit is available at https://github.com/espnet/espnet/tree/master/egs2/TEMPLATE/asr1/pyscripts/utils, accessed on 7 July 2023.
